# Conserved missense variant in *ALDH1A3* ortholog impairs fecundity in *C. elegans*

**DOI:** 10.17912/micropub.biology.000357

**Published:** 2021-01-14

**Authors:** Wan-Rong Wong, Shayda Maher, Jun Young Oh, Katherine I Brugman, Shahla Gharib, Paul W Sternberg

**Affiliations:** 1 Division of Biology and Biological Engineering, California Institute of Technology; 2 Department of Neurobiology, Northwestern University

## Abstract

Accumulating evidence demonstrates that mutations in *ALDH1A3 *(the aldehyde dehydrogenase 1 family, member A3) are associated with developmental defects. The ALDH1A3 enzyme catalyzes retinoic acid biosynthesis and is essential to patterning and neuronal differentiation in the development of embryonic nervous system. Several missense mutations in *ALDH1A3 *have been identified in family studies of autosomal recessive microphthalmia, autism spectrum disorder, and other neurological disorders. However, there has been no evidence from animal models that verify the functional consequence of missense mutations in *ALDH1A3*. Here, we introduced the equivalent of the *ALDH1A3 *C174Y variant into the *Caenorhabditis elegans *ortholog, *alh-1*, at the corresponding locus. Mutant animals with this missense mutation exhibited decreased fecundity by 50% compared to wild-type animals, indicating disrupted protein function. To our knowledge, this is the first ALDH1A3 C174Y missense model, which might be used to elucidate the effects of ALDH1A3 C174Y missense mutation in the retinoic acid signaling pathway during development.

**Figure 1. The ALDH1A3(C174Y) variant and its C. elegans ortholog, alh-1(C172Y):**
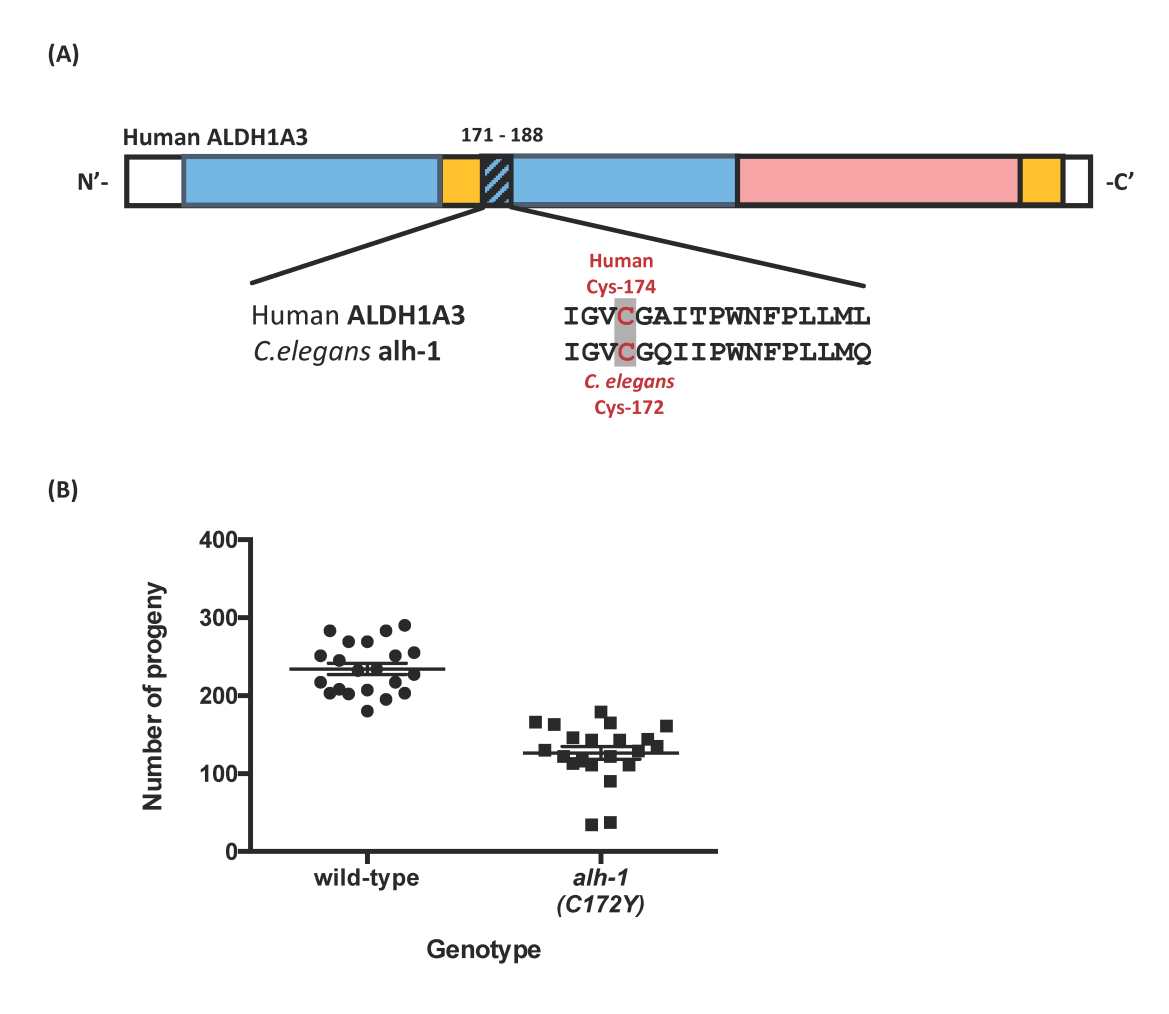
**(A)** Domains and sequence conservation between human *ALDH1A3* and its *C. elegans* ortholog, *alh-1*. The three domains in human ALDH1A3 are colored differently: the NAD binding domain is shown in blue, the catalytic domain in pink, and the oligomerization domain in yellow. Overall, the human ALDH1A3 is conserved with *C. elegans* ALH-1 at 77% of amino acid positions. The protein sequence alignment around the ALDH1A3 C174Y residue is highlighted in the bottom row. **(B)** In our *C. elegans* model, the *alh-1 (C172Y)* missense mutant displayed a significantly reduced fecundity. Approximately 20 animals were tested in each strain. Each dot represents one hermaphrodite. *P*< 0.0001 via non-parametric Mann-Whitney test.

## Description

Retinoic acid (RA), the active metabolite of vitamin A, broadly regulates gene expression. The RA signaling pathway plays an essential role in embryonic development, including the development of the body axis, eye, brain, and heart (Ghyselinck & Duester, 2019). One of the key enzymes in the biosynthesis of RA is aldehyde dehydrogenase 1 family member A3 (ALDH1A3). ALDH1A3 converts retinaldehyde to retinoic acid and it is expressed early in forebrain development (McCaffery & Drager, 1994). Several mutations in *ALDH1A3* have been implicated in patients with autosomal recessive microphthalmia and other neurological disorders (Fares-Taie *et al.*, 2013; Roos *et al.*, 2014). However, current animal models of ALDH1A3 have large truncations of the protein. Direct evidence of the effects of missense variants on ALDH1A3 protein activity has not yet been obtained.

In this study, we aimed to determine the functional consequence of *ALDH1A3(C174Y)* missense variants implicated in patients (Roos *et al.*, 2014). Patients with the Cys174Tyr missense variant presented with autosomal recessive microphthalmia, autistic symptoms, and intellectual disabilities. The human ALDH1A3 C174Y missense residue is located at the amino-terminal nicotinamide adenine dinucleotide (NAD)-binding domain (Moretti *et al.*, 2016), which is evolutionarily conserved between human and commonly used model organisms, such as *C. elegans*, *Drosophila,* zebrafish, and mouse. The *ALDH1A3* orthologs in these model organisms have similar amino acids at >75% of positions of the human ALDH1A3 protein. We believe the conservation of protein sequences among various species indicates the essential role of this domain in protein functions. Here, we use *C. elegans* as a prime candidate for studying the causality of missense variants in *ALDH1A3*. We generated the targeted *ALDH1A3 C174Y* missense variant in its *C. elegans* orthologous gene *alh-1* (Figure 1A).

The short lifespan and genetically modifiable nature of *C. elegans* allow rapid screening of functional impactful missense mutation implicated in human diseases. We previously established a working pipeline to introduce autism-associated missense mutations into the genome of *C. elegans* using CRISPR/Cas9 and homology-directed repair (Wong *et al.*, 2019). Here, we used this pipeline to generate a missense mutant, *alh-1 (C172Y)*, that matches the human *ALDH1A3(C174Y)* variant. We then characterized this *C. elegans* missense mutant in a fecundity assay to quantify the robustness of germline development. The *C. elegans* germline development is a highly sensitive process, which reflects subtle changes in sensory perception, feeding behavior, and metabolism (Hubbard *et al.*, 2013). As a result, we used the *C. elegans* fecundity assay as a screening tool to identify functional changing missense mutations, as defects in germline development will result in a decreased brood size.

We evaluated the impact of C172Y missense mutation on development using a fecundity assay, which measures the number of viable progeny per hermaphrodite. The *alh-1(C172Y)* missense mutant nematode, *alh-1(sy899)*, showed only 54% of wild-type fecundity (Figure 1B. Wild-type: 234 ± 7; *alh-1(C172Y)*: 127 ± 8. *p* < 0.0001). We observed that two of the eighteen mutant animals had very low brood size and a lot of unhatched eggs (17 unhatched eggs to a brood size of 34, and 4 unhatched eggs to a brood size of 38). The other animals had essentially no unhatched eggs or dead larvae. This result indicated a malfunction in the aldehyde dehydrogenase protein, resulting in partial sterile and germline developmental defects. Our result is consistent with the observation that the *C. elegans alh-1(tm5823)* putative null mutant strain produces some dead animals and a highly expressed sterile phenotype (personal communication with Dr. Shohei Mitani, NBPJ). The *alh-1(tm5823)* mutant harbors a 618 bp deletion, located outside of our C172Y missense site and removes part of the NAD-binding and catalytic domains. Our conserved C172Y missense residue is also located in the NAD-binding domain. In addition, the developmental phenotype is displayed in other animal models of the aldehyde dehydrogenase family. For example, *Aldh* null larvae and adults are less viable in the presence of ethanol in *Drosophila* (Fry & Saweikis, 2006). Moreover, mouse *Aldh3^-/- ^*homozygotes were impaired in early forebrain development, possibly through failure to induce genes needed to establish regionalization (Molotkova *et al.*, 2007). The *Aldh1a3* null mouse showed perinatal lethality that could be rescued by maternal RA treatment (Dupé *et al.*, 2003).

The developmental defects caused by mutations in *ALDH1A3* are likely to act through alteration in the RA signaling pathway. RA binds to its nuclear RA receptor (RAR), which forms a heterodimer with retinoid X receptors (RXRs) and RA response elements (RARE). The RAR/RXR/RARE complex regulates gene transcription at specific locations during various developmental stages. In addition, RA is known to promote the differentiation of neurons prenatally; RA concentration, indicated indirectly by ALDH1A3 expression, influences the maturation of selected parts of the cerebral cortex postnatally (Wagner *et al.*, 2006). This could be a possible explanation of a connection between *ALDH1A3* mutations and the neurological symptoms displayed in patients with intellectual disabilities and autism spectrum disorder. Our result illustrated that ALH-1(C172Y) residue is necessary for wild-type ALH-1 protein function in *C. elegans*. Since the ALH-1(C172Y) residue is conserved between *C. elegans* and humans, it is likely that this residue also has a functionally significant role in human ALDH1A3 protein. Further study in other model organisms is needed to validate this and potentially elucidate the mechanistic basis of the functional consequences of the *ALDH1A3(C174Y)* variation.

## Methods

Fecundity assay

Well-fed *C. elegans* were synchronized at the L4 stage. In the fecundity assay, individual L4 hermaphrodites were placed on separate NGM plates seeded with OP50, and these animals were subsequently transferred to a new plate every day. The number of newly hatched larvae progeny was counted for every plate 1 day after the adult was transferred. The total fecundity comprises the sum of progeny produced for four days per animal.

Statistical analysis

The fecundity assay was analyzed by Mann-Whitney test using GraphPad Prism version 6 (GraphPad, La Jolla, CA).

## Reagents

The Bristol N2 *C. elegans* strain was used as the wild-type control and background for the CRISPR experiments (Brenner, 1974). The *alh-1(C172Y)* missense strain, PS7481 *alh-1(sy899)*, was generated using the protocol described previously (Wong *et al.*, 2019). Detection primers sequence: forward primer 5’-TACAGTTATTACGCCGGATGG-3’; reverse primer 5’- CATGTGCGACGAAATAGCTTG -3’. The expected PCR product length for the wild-type is 370 bp; PCR product from mutant strain can be further digested by HaeIII (New England Biolabs, Ipswich, MA) into two fragments of 104 and 266 bp. All strains were maintained on nematode growth medium (NGM) agar plates seeded with *Escherichia coli* OP50 at room temperature (21 ± 1^◦^C).
